# Supplementary material for: MXRA8-dependent chikungunya virus infection in PDGFRα+ mesenchymal cells increases vascular permeability and facilitates hematogenous spread
Source: bioRxiv. 2026 Oct 7:2026.10.06.757040. Preprint. [Version 1] doi: 10.64898/2026.10.06.757040 (PMC13692254; doi:10.64898/2026.10.06.757040)
Supplement: Supplementary file 1 [file NIHPP2026.10.06.757040v1-supplement-1.pdf]

bioRxiv preprint doi: https://doi.org/10.64898/2026.10.06.757040; this version posted October 8, 2026. The copyright holder for this preprint (which was not certified by peer review) is the author/funder, who has granted bioRxiv a license to display the preprint in perpetuity. It is made available under a CC-BY 4.0 International license.

SUPPLEMENTAL FIGURE LEGENDS

Figure S1. Flow cytometry gating scheme for CHIKV-infected cells. Non-hematopoietic cells were defined as live, single, CD45^{-} cells, and hematopoietic cells were defined as live, single, CD45^{+} cells. Both cell compartments were analyzed for YFP expression after CHIKV-AF15561-Venus infection. Naive cells were used to set gates. Monocytes: live, single, CD45^{+}, CD11b^{+}, Ly6C^{hi} cells; endothelial cells: live, single, CD45^{-}, CD31^{+} cells; FAPs: live, single, CD31$^{-,}$ CD29^{+}, Sca-1^{+} cells; and pericytes: live, single, CD45^{-}, CD31^{-}, CD29^{-}, CD146^{+} cells. FAPs were analyzed for intracellular VEGF-A expression and endothelial cells were analyzed for VEGFR-2 expression.

Figure S2. Genotyping of *Pdgfra-Cre*^{ERT2} *Mxra8*$^{fl/fl}$ mice. (**A**) Location of the *loxP* sites flanking exons 2-9 of the mouse *Mxra8* gene (*top panel*) and location of the primer binding sites for primers used to validate *Pdgfra-Cre*^{ERT2} *Mxra8*$^{fl/fl}$ mice. (**B**) Animals were administered tamoxifen (Tam) as indicated in the Methods. Tail sections were clipped from two separate *Pdgfra-Cre*$^{ERT2+}$ or Cre- mice and digested in lysis buffer. Genomic DNA was isolated and used as a template in three separate PCR reactions. Excised and floxed regions of the *Mxra8* gene were amplified by PCR using the following primers: Recombination product (lanes 3-6), Forward 5' GTCCTGCTGTGGAAACTGCTGC 3' and Reverse 5' CTTGATTCCCAGCTGACTCGCC; Floxed product (lanes 7-10) Forward 5' GTCCTGCTATGGAAACTGCTAC 3' and Reverse 5' CTTGATTCCCAGCTGACTCGCC. *Pdgfra-Cre*^{ERT2} (lanes 11-14) was amplified using the following primers: Forward 5' ATCGCATTCCTTGCAAAAGT 3' and Reverse 5' AGGCCCACAGAACATGGAC 3'.

Figure S3. MAYV infection of *Pdgfra-Cre*^{ERT2} *Mxra8*$^{fl/fl}$ mice. (**A**) Three-week-old male and female *Pdgfra-Cre*^{ERT2} *Mxra8*$^{fl/fl}$ mice were administered tamoxifen (Tam) every other day for 5 days via intraperitoneal injection. Subsequently, *Pdgfra-Cre*$^{ERT2+}$ or Cre- *Mxra8*$^{fl/fl}$ mice (n = 7-9 mice per group, 2 experiments) were inoculated with 10^3 FFU of MAYV via subcutaneous footpad injection. (**B**) At 3 dpi, footpad swelling was measured. Mice were then sacrificed,

bioRxiv preprint doi: https://doi.org/10.64898/2026.10.06.757040; this version posted October 8, 2026. The copyright holder for this preprint (which was not certified by peer review) is the author/funder, who has granted bioRxiv a license to display the preprint in perpetuity. It is made available under a CC-BY 4.0 International license.

perfused with PBS, and ipsilateral foot (**C**), ipsilateral calf muscle (**D**), contralateral foot (**E**), and contralateral calf muscle (**F**) were harvested. Viral RNA in each tissue was measured by qRT-PCR. Statistical analysis: (**B-F**) Mann-Whitney test; *P** < 0.05, ***P* < 0.01, ****P* < 0.001, ns, not significant. Dotted horizontal lines on graphs indicate limit of detection for the assay.

Figure S4. CHIKV infection of *Vav1-Cre Mxra8*$^{fl/fl}$ with CHIKV-AF or CHIKV-AF-Venus. (**A**) Tail sections were clipped or blood was collected from two separate *Vav1-Cre Mxra8*$^{fl/fl}$ mice. Tails or whole blood were digested in lysis buffer. Genomic DNA was isolated and used as a template in two separate PCR reactions to amplify products of *Mxra8* gene excision (*top panel*) or *Vav1*-Cre (*bottom panel*). (**B-F**) Four-week-old male and female *Vav1-Cre Mxra8*$^{fl/fl}$ mice (n = 6-10 mice per group, 2 experiments) were inoculated with 10^3 FFU of CHIKV-AF via subcutaneous footpad injection. At 3 dpi, footpad swelling (**B**) was measured. Mice were then sacrificed, perfused with PBS, and the ipsilateral foot (**C**), ipsilateral calf muscle (**D**), contralateral foot (**E**), and contralateral calf muscle (**F**) were harvested. Viral RNA in each tissue was measured. (**G-I**). Cre- or *Vav1*-Cre *Mxra8*$^{fl/fl}$ mice were inoculated with 10^5 FFU of CHIKV-AF-Venus in the footpad. At 2 dpi, ipsilateral feet were collected and processed as in **Fig 1 and 2**. The percentage (**G**) and number of FAPs (**H**) or endothelial cells (**I**) expressing YFP were quantified by flow cytometry. Statistical analysis: (**B-I**) Mann-Whitney test; ns, not significant. Dotted horizontal lines on graphs indicate limit of detection for the assay.

Figure S5. MXRA8 expression in fibroblasts and endothelial cells isolated from the feet of *Pdgfra-Cre*^{ERT2} *Mxra8*$^{fl/fl}$ mice. (**A**) Three-week-old male and female Cre- or *Pdgfra-Cre*^{ERT2} *Mxra8*$^{fl/fl}$ mice were administered tamoxifen (Tam) every other day for 5 days via intraperitoneal injection. Both feet were harvested and processed as in **Fig 1, 2, and S3**. Cells were pooled from 8 Cre+ or 8 Cre- mice, and the experiment was repeated four times. CD45$^-$ cells were sorted into the CD31$^+$ endothelial cells and CD31$^-$ CD29$^+$ fibroblast fractions. (**B**) Cellular mRNA was extracted from 10^5 endothelial cells or fibroblasts to measure *Mxra8* mRNA levels by qRT-PCR and was normalized to *Gapdh* mRNA levels. (**C**) In a separate experiment,

bioRxiv preprint doi: https://doi.org/10.64898/2026.10.06.757040; this version posted October 8, 2026. The copyright holder for this preprint (which was not certified by peer review) is the author/funder, who has granted bioRxiv a license to display the preprint in perpetuity. It is made available under a CC-BY 4.0 International license.

DNA was extracted from 10^5 endothelial cells or fibroblasts and used a template to amplify regions of the genome corresponding to products of Cre-recombinase-mediated excision. (**D-E**) Processed single-cell RNA-seq data (count matrix and cell metadata) were downloaded from GEO accession GSE300299 [36]. (n = 3 per group; [70]). Statistical analysis: Mann-Whitney test; **P* < 0.05. Dotted line indicates limit of detection for the assay.

Figure S6. ISG expression and IFNAR1 blockade. (**A**) RNA was extracted from 2 x 10^5 NIH 3T3 fibroblasts or SVEC4-10 cells and used as template to measure mRNA levels of a panel of genes (*Ifna*, *Ifnb*, *Il1b*, *Il6*, *Oas1a*, and *Ifit1*) by qRT-PCR. *Gapdh* levels were used to normalize between cell types. (**B**) Primary fibroblasts and endothelial cells were isolated, pooled, and sorted from both feet of WT mice (n = 7), and levels of *Oas1a* mRNA were measured by qRT-PCR. (**C**) SVEC4-10 cells were pre-treated with anti-IFNAR1 mAb (MAR1-5A3) or isotype control antibodies overnight at 37°C. Treated cells were then inoculated with CHIKV-AF at a MOI of 1 and collected at 24 hpi for analysis of intracellular E2 expression as described in **Fig 3**. Statistical analysis: Two-way ANOVA test with Šídák's post-test; **P* < 0.05 *****P* < 0.0001. Dotted horizontal lines on graphs indicate limit of detection for the assay.

Figure S7. Number of FAPs and endothelial cells isolated from mock and CHIKV-infected mice. Cohorts of 4-week-old C57BL6/J WT, *Mxra8*$^{\Delta 8/\Delta 8}$, tamoxifen (Tam)-treated *Pdgfra-Cre*^{ERT2}*, Mxra8*$^{fl/fl}$, or Tam-treated Cre- mice (n = 6 mice per group, 2 experiments) were inoculated with 10^3 FFU of CHIKV-AF in the left rear footpad or mock-infected. At 3 dpi, mice were administered brefeldin A via intraperitoneal injection 6 h prior to sacrifice. Ipsilateral feet were harvested and digested to release stromal cells. Cell suspensions were incubated with anti-CD45, anti-CD31, anti-CD29, anti-SCA1, anti-VEGFR-2, and anti-VEGF-A antibodies, and the total number of FAPs (**A**) and endothelial cells (**B**) were determined by flow cytometry. Statistical analysis: Two-way ANOVA test with Tukey's post-test; ns, not significant.

bioRxiv preprint doi: https://doi.org/10.64898/2026.10.06.757040; this version posted October 8, 2026. The copyright holder for this preprint (which was not certified by peer review) is the author/funder, who has granted bioRxiv a license to display the preprint in perpetuity. It is made available under a CC-BY 4.0 International license.

Figure S1

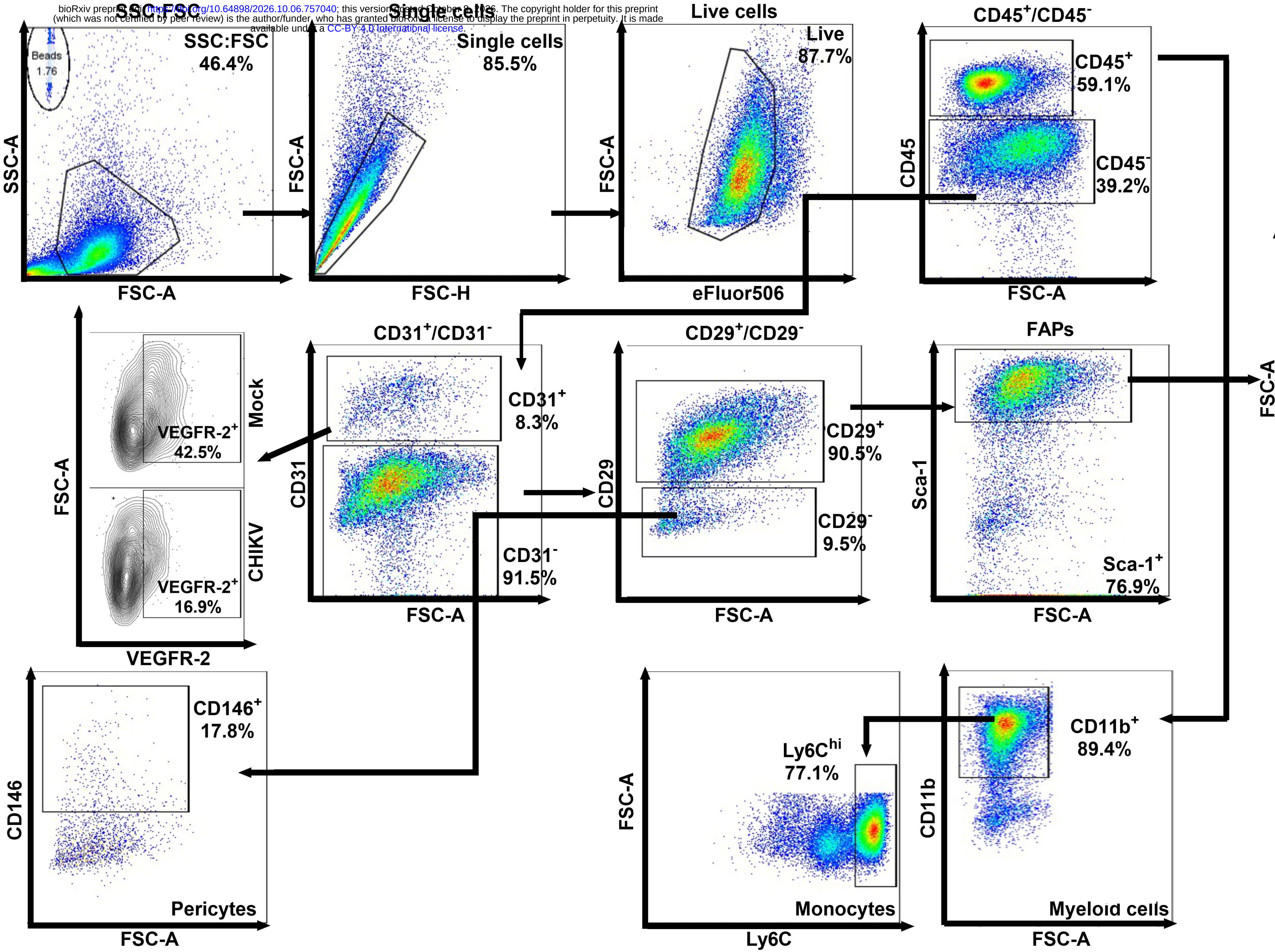

Figure S2

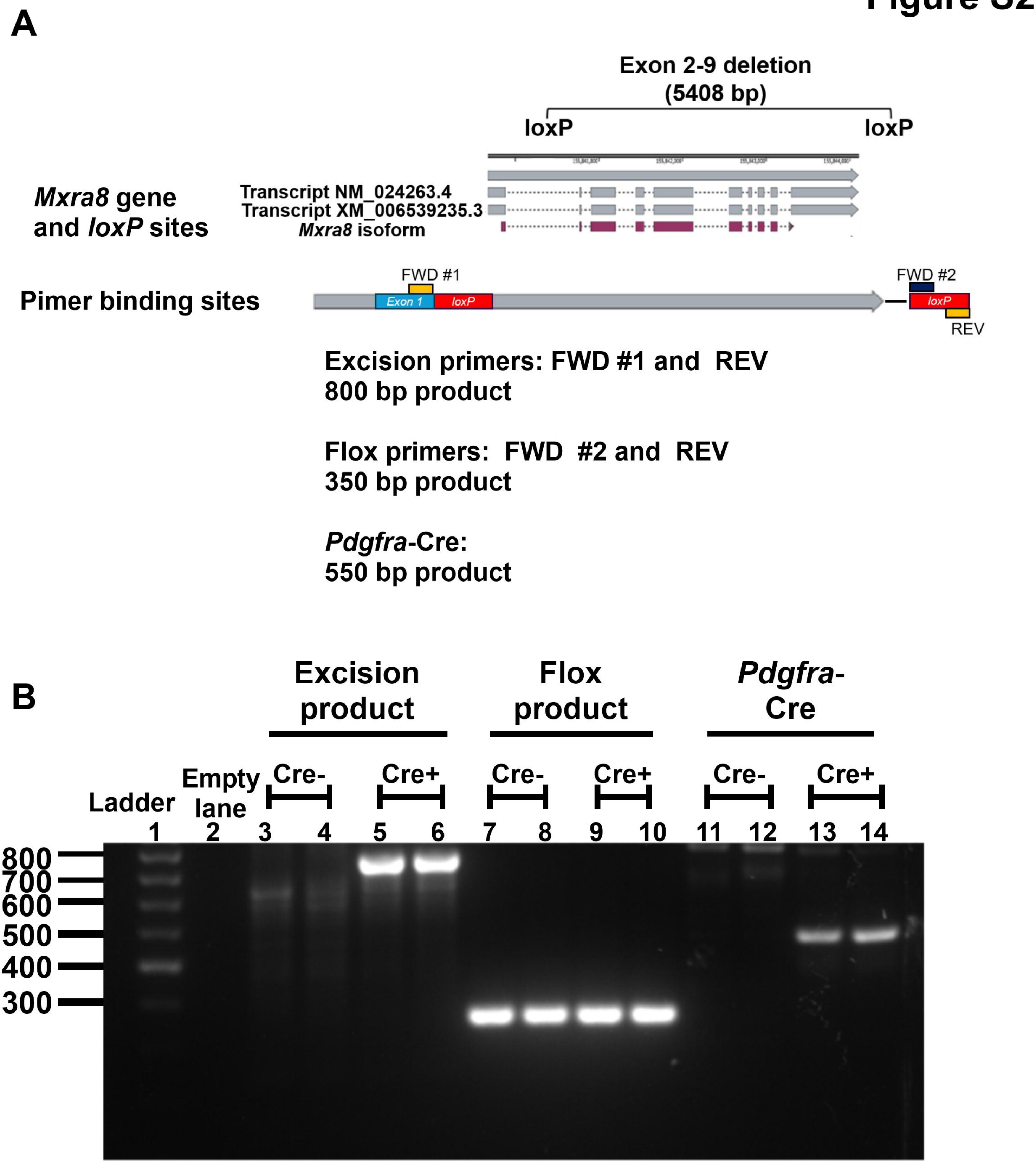
A
Exon 2-9 deletion
(5408 bp)
loxP
loxP
Mxra8 gene and loxP sites
Transcript NM_024263.4
Transcript XM_006539235.3
Mxra8 isoform
Pimer binding sites
FWD #1
Exon 1
loxP
FWD #2
loxP
REV
Excision primers: FWD #1 and REV
800 bp product
Flox primers: FWD #2 and REV
350 bp product
Pdgfra-Cre:
550 bp product
B
Excision product
Flox product
Pdgfra-Cre
Ladder
Empty lane
Cre-
Cre+
Cre-
Cre+
Cre-
Cre+
1 2 3 4 5 6 7 8 9 10 11 12 13 14
800
700
600
500
400
300

Figure S3

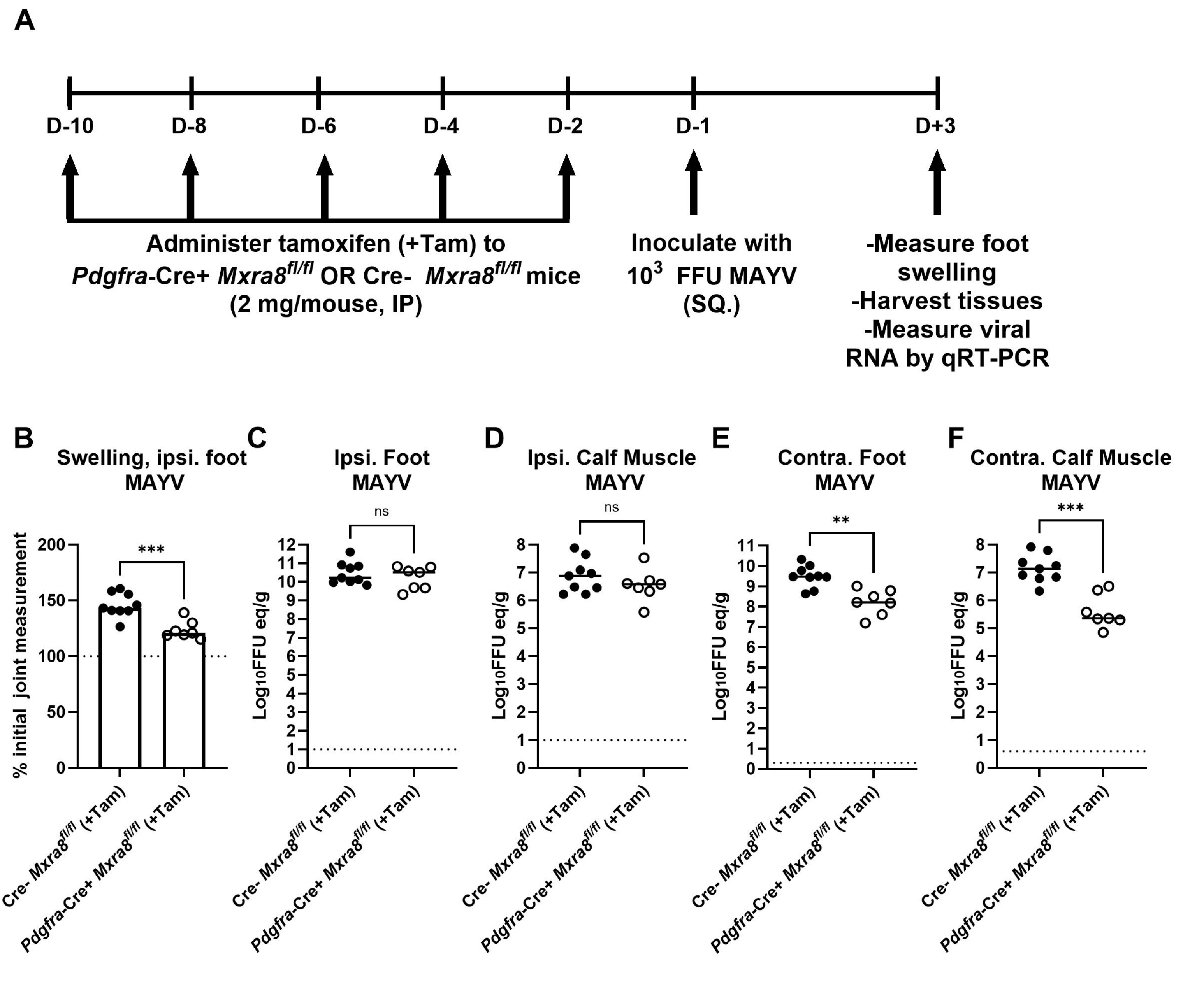
A
D-10
D-8
D-6
D-4
D-2
D-1
D+3
Administer tamoxifen (+Tam) to
Pdgfra-Cre+ Mxra8fl/fl OR Cre- Mxra8fl/fl mice
(2 mg/mouse, IP)
Inoculate with
10³ FFU MAYV
(SQ.)
-Measure foot
swelling
-Harvest tissues
-Measure viral
RNA by qRT-PCR
B
Swelling, ipsi. foot
MAYV
% initial joint measurement
***
C
Ipsi. Foot
MAYV
ns
D
Ipsi. Calf Muscle
MAYV
ns
E
Contra. Foot
MAYV
**
F
Contra. Calf Muscle
MAYV
***
Log10FFU eq/g
Cre- Mxra8fl/fl (+Tam)
Pdgfra-Cre+ Mxra8fl/fl (+Tam)

bioRxiv preprint doi: https://doi.org/10.64898/2026.10.06.757040; this version posted October 8, 2026. The copyright holder for this preprint (which was not certified by peer review) is the author/funder, who has granted bioRxiv a license to display the preprint in perpetuity. It is made available under a CC-BY 4.0 International license.

Figure S4

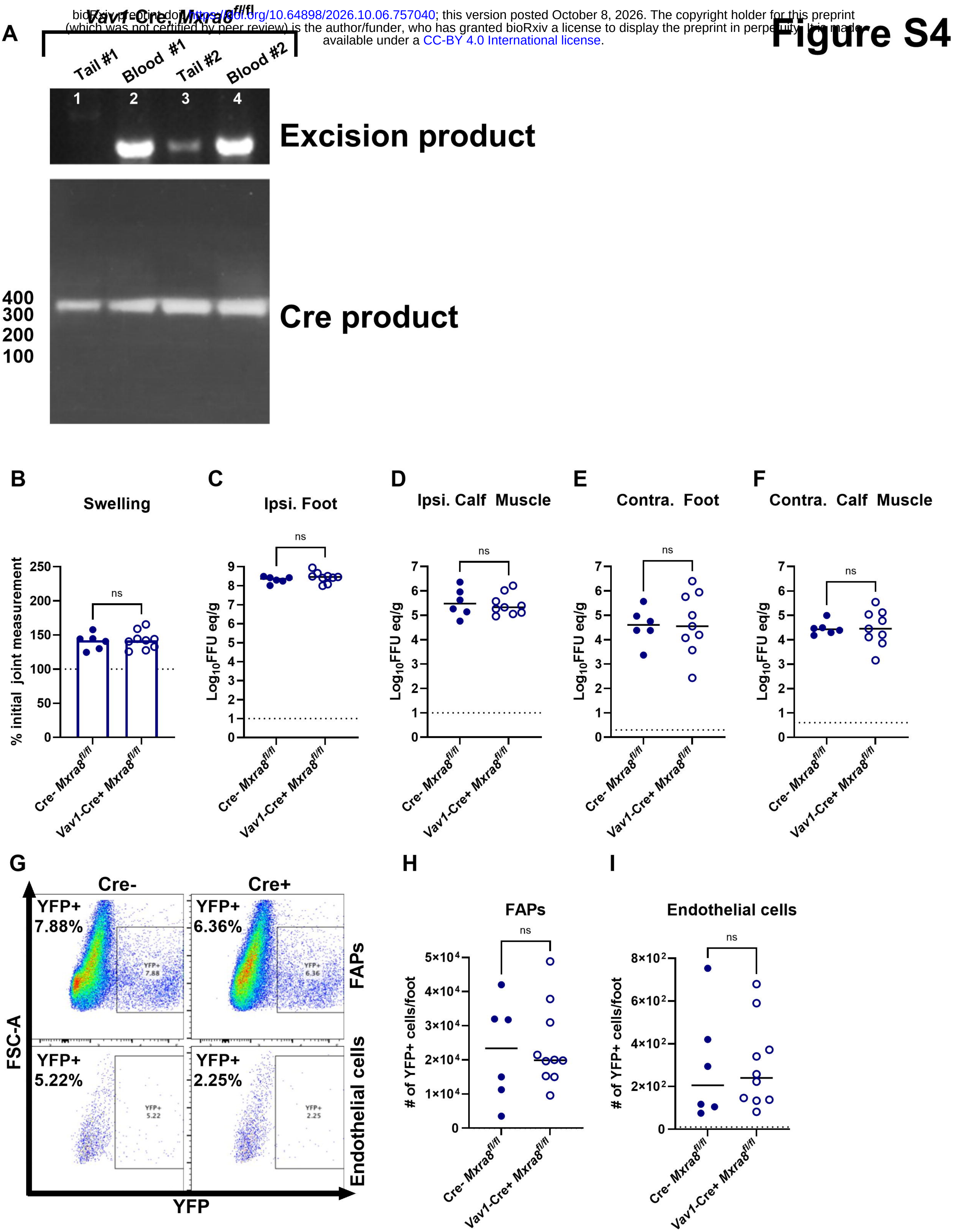

Figure S5

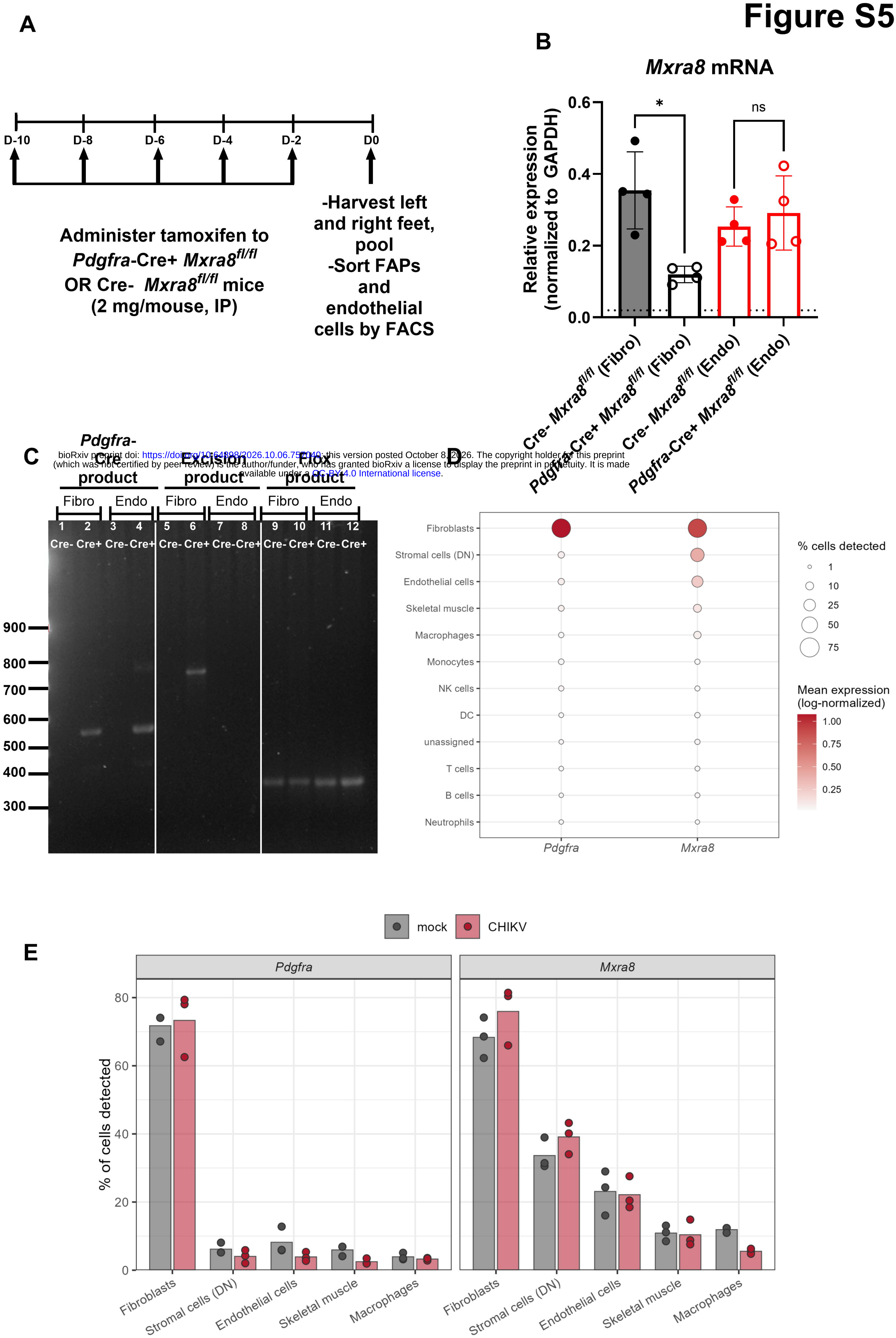

bioRxiv preprint doi: https://doi.org/10.64898/2026.10.06.757040; this version posted October 8, 2026. The copyright holder for this preprint (which was not certified by peer review) is the author/funder, who has granted bioRxiv a license to display the preprint in perpetuity. It is made available under a CC-BY 4.0 International license.

Figure S6

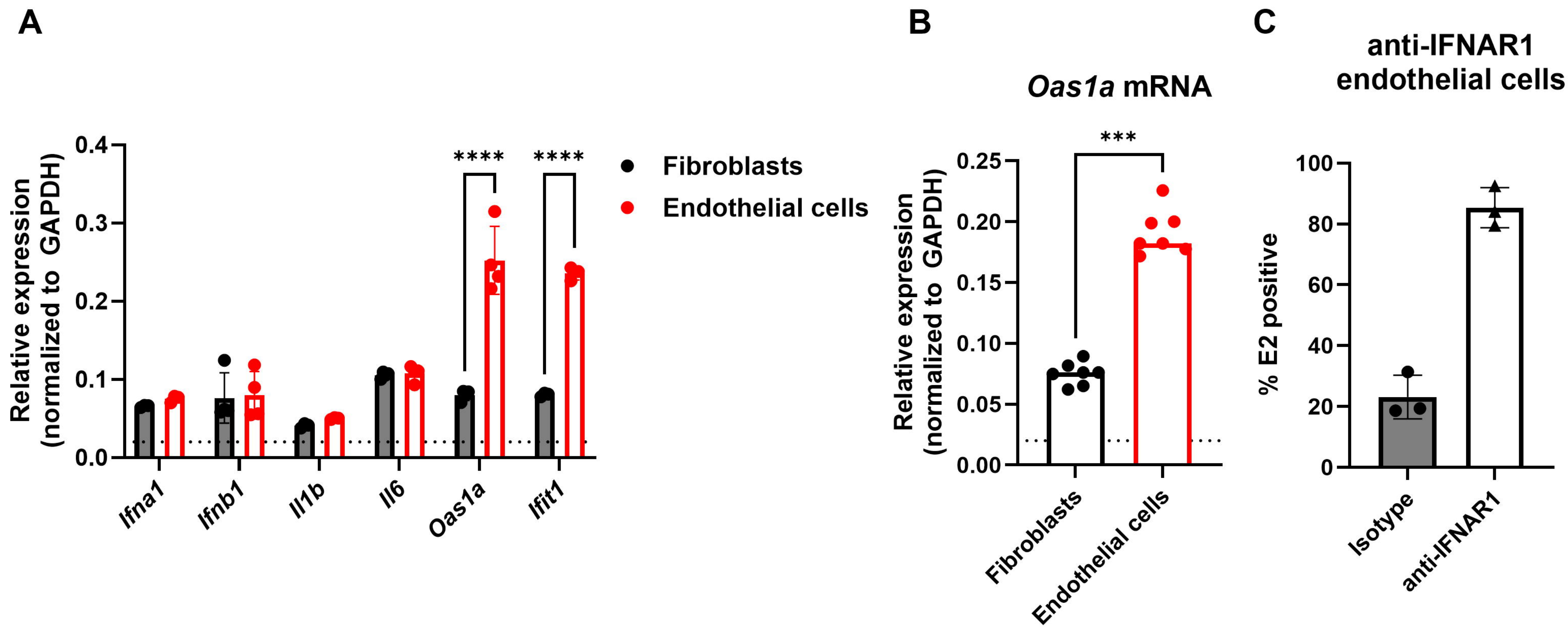

A
Relative expression (normalized to GAPDH)
0.0
0.1
0.2
0.3
0.4
****
****
Fibroblasts
Endothelial cells
Ifna1
Ifnb1
Il1b
Il6
Oas1a
Ifit1
B
Oas1a mRNA
***
0.00
0.05
0.10
0.15
0.20
0.25
Fibroblasts
Endothelial cells
C
anti-IFNAR1 endothelial cells
% E2 positive
0
20
40
60
80
100
Isotype
anti-IFNAR1

Figure S7

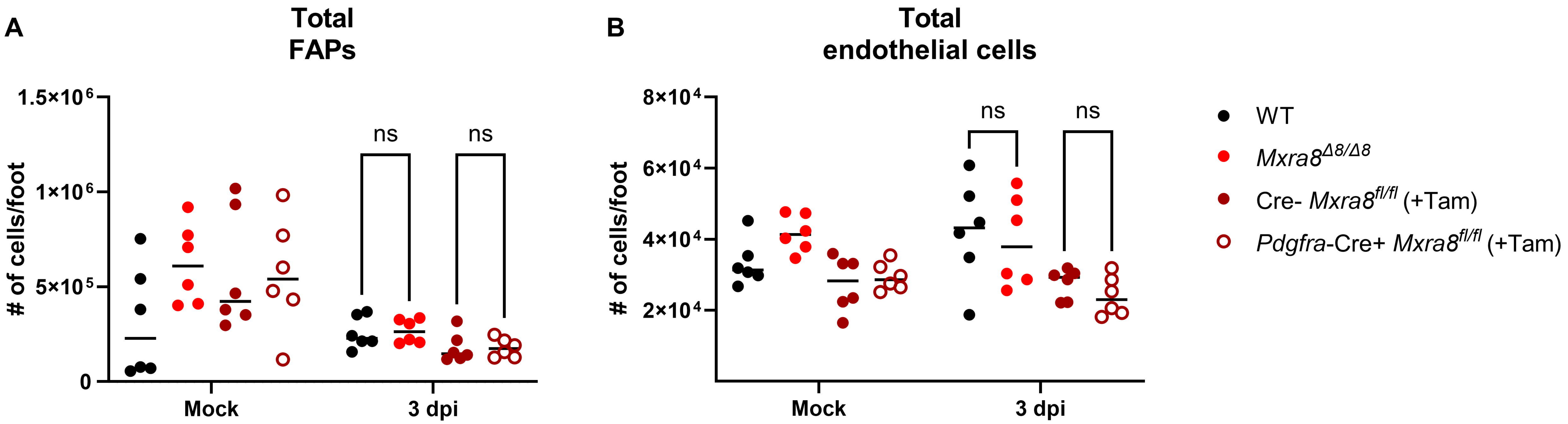
A
Total
FAPs
1.5×10^6
1×10^6
5×10^5
0
# of cells/foot
ns
ns
Mock
3 dpi
B
Total
endothelial cells
8×10^4
6×10^4
4×10^4
2×10^4
# of cells/foot
ns
ns
Mock
3 dpi
WT
Mxra8^Δ8/Δ8
Cre- Mxra8^fl/fl (+Tam)
Pdgfra-Cre+ Mxra8^fl/fl (+Tam)
